# Transmission of haemotropic mycoplasma in the absence of arthropod vectors within a closed population of dogs on ectoparasiticides

**DOI:** 10.1038/s41598-023-37079-z

**Published:** 2023-06-22

**Authors:** Lucas G. Huggins, Zahida Baydoun, Ron Mab, Yulia Khouri, Bettina Schunack, Rebecca J. Traub, Vito Colella

**Affiliations:** 1 Melbourne Veterinary School, Faculty of Science, University of Melbourne, Parkville, VIC 3050 Australia; 2 Animal Mama Veterinary Hospital, Phnom Penh, 12312 Cambodia; 3 Elanco GmbH, Heinz-Lohmann-Str. 4, 27472 Cuxhaven, Germany

**Keywords:** Pathogens, Parasite biology, Infectious-disease epidemiology, Applied microbiology

## Abstract

Dog-infecting haemotropic mycoplasmas (haemoplasmas), such as *Mycoplasma* *haemocanis* and *Candidatus* Mycoplasma haematoparvum are common blood-borne pathogens of canines that can potentially inflict a substantial burden of disease, particularly in immunosuppressed individuals. Nonetheless, the transmission of these pathogens remains debated as more evidence emerges that they may not be transmitted by vectors, but instead use alternative methods such as aggressive interactions and vertical transmission. Here, we treated forty dogs with two different topically-acting ectoparasiticide products able to prevent vector-borne pathogen infections during an 8-month community trial in Cambodia. A total absence of ectoparasites were observed at all time points, and no new infections caused by pathogens confirmed as being vectorially-transmitted were detected, i.e., *Babesia* *vogeli*, *Ehrlichia* *canis*, *Anaplasma* *platys*, and *Hepatozoon* *canis*. Conversely, the number of haemoplasma infections in dogs on both ectoparasiticides rose significantly, with an incidence of 26 infections per 100 dogs at risk per year, providing strong evidence of non-vectorial transmission. Over the study period, dog aggression and fighting were frequently observed, highlighting a different potential mode of transmission. This study presents the first robust evidence that canine haemoplasmas may be transmitted without arthropod vectors drawing attention to the need for new methods to prevent their transmission.

## Introduction

Haemotropic mycoplasmas alternatively known as haemoplasmas are small epicellular bacterial pathogens, that typically reside on the surface of red blood cells^1–3^. These organisms lack a cell wall, are to date non-culturable and typically exhibit reduced genome sizes due to their parasitic existence^4, 5^. Haemotropic mycoplasmas can be relatively common blood-borne pathogens of canines that have been found across the globe, with species such as *Mycoplasma* *haemocanis* and *Candidatus* Mycoplasma haematoparvum being some of the most frequently detected^2, 3, 6–9^. These species can cause haemolytic anaemia which can range from a condition involving severe pathology, particularly in splenectomised or immunosuppressed dogs, through to a chronic condition with an absence of clinical signs that may go undetected^3, 7, 10–13^. In addition, the DNA of other haemotropic mycoplasmas has also been identified from dog blood, including *Candidatus* Mycoplasma turicensis^14, 15^, *Candidatus* Mycoplasma haemobos^5, 9^, *Candidatus* Mycoplasma haematominutum^16^ and an undescribed species infecting dogs in Australia and Cambodia^12, 13, 17^.

Importantly, some canine-infecting haemoplasma species have been found to cause infections in humans, e.g., *C.* M. haematoparvum which has been identified from a veterinarian exhibiting neurological symptoms including seizures and extreme headaches, raising questions on such species zoonotic potential^18^. Similarly, other zoonotic but non-canine haemotropic mycoplasma species have also been identified as generating human infections, for instance *Candidatus* Mycoplasma haemohominis^19–21^, a *Mycoplasma* *ovis*-like bacteria^22, 23^, and a *Mycoplasma* *suis*-like bacteria^24^. The clinical presentation in humans infected by these species is varied but in the case of *C.* M. haemohominis spans a range of symptoms including persistent pyrexia, haemolytic anaemia, and pancytopenia^19, 20^.

The transmission of canine haemotropic mycoplasmas remains a hotly debated topic that is exacerbated by them being unable to be axenically cultured^1–4, 25^. This characteristic of haemoplasmas consequently hampers researchers’ ability to conduct conclusive transmission studies and leads to a dearth in data on their basic biology^2^. Early investigations into canine haemotropic mycoplasmas suggested that transmission may be primarily facilitated using blood-feeding arthropods, such as the brown dog tick, *Rhipicephalus* *sanguineus* sensu lato^26^*,* more specifically the “tropical lineage” recently recognized as *Rhipicephalus* *linnaei*^27, 28^. Since then, other authors have highlighted vectors, including ticks and fleas, as likely taking a primary role in transmission^7, 29^. This is supported by more concrete evidence from studies exploring the transmission of the feline-infecting species *Mycoplasma* *haemofelis*, a close phylogenetic relative of *M.* *haemocanis*, where the role of *Ctenocephalides* *felis* fleas is strongly implicated in transmission^30^. Nevertheless, numerous studies have shown no association between the presence of arthropod vectors and haemoplasmas^31–33^ and other transmission modes have been reported, such as through blood transfusion^34^, vertical transmission from bitch-to-pup^35^, fighting, and social contact^33, 36, 37^. The detection of DNA from some feline haemoplasma species within salivary secretions of infected cats, highlights another mode by which these organisms may be transmitted^37, 38^. Therefore, whether multiple transmission modes are used by canine haemotropic mycoplasmas concurrently, or if different species use different forms of transmission, is a question that urgently needs addressing.

Here, we set out to assess whether two different topically-acting ectoparasiticide products would be able to prevent ectoparasite infestation and VBPs transmission during an 8-month long community trial conducted on 40 dogs in Cambodia. These topically-acting products act from the skin and fur of the animal to be quickly lethal and/or repel ectoparasites that might bite and transmit VBPs and elucidation of their efficacy in such a high VBP-transmission pressure context is crucial data for veterinarians and pet owners within the region^39, 40^.

## Results

Of the forty dogs investigated (cohort composition in Table 1) none were found with ectoparasites attached or present within either group at all timepoints, including baseline, demonstrating the efficacy of both Detick and Seresto^®^ at preventing infestation by arthropod vectors.

**Table 1:** Canine sex, age, breed, and neutering status in the Seresto^®^ group and Detick group. Cross breed refers to local mongrel dogs that do not have obvious recognisable characteristics of a common dog breed.

	Seresto^®^ group	Detick group	Total
Sex			
Female	11 (55%)	12 (60%)	23
Male	9 (45%)	8 (40%)	17
Total	20	20	40
Age			
> 6 months < 12 months	5 (25%)	5 (25%)	10
> 12 months	13 (65%)	15 (75%)	28
Unreported	2 (10%)	0 (0%)	2
Total	20	20	40
Neutered			
Yes	14 (70%)	20 (100%)	34
No	6 (30%)	0 (0%)	6
Total	20	20	40
Breed			
Cross	19 (95%)	10 (50%)	29
Pure	0 (0%)	1 (5%)	1
Unreported	1 (5%)	9 (45%)	10
Total	20	20	40

Only qPCR was able to detect any of the identified blood-borne pathogens throughout this study with no infections found via microscopic examination of stained whole blood and buffy coat smears. The number of blood-borne pathogen infections detected via qPCR was highest at baseline (Table 2), i.e., prior to Detick and Seresto^®^ application. Following on from baseline assessment the only newly acquired blood-borne pathogen infections were haemotropic mycoplasmas, with no other infections identified apart from one individual that remained *Hepatozoon* *canis* positive from baseline to the end of the study (Table 2). Given the challenges associated with *H.* *canis* curative treatment^41^ and because the dog did not show clinical signs throughout the study period, it was decided to withhold treatment, resulting in the subject continuing to remain positive till the end of the study period. When the number of infections for both groups of dogs were combined, no significant difference between haemotropic mycoplasma infections at baseline were observed, when compared to confirmed VBP infections, i.e., *A.* *platys*, *E.* *canis* and *H.* *canis* (χ^2^ test statistic 0.157, p = 0.69). However, after the application of either ectoparasiticide the number of newly acquired haemotropic mycoplasma infections was significantly higher than new infections by a pathogen known to be transmitted by vectors, at every time-point; 3-months (χ^2^ test statistic 5.000, p = 0.025), 6-months (χ^2^ test statistic 9.804, p = 0.002), and 8-months (χ^2^ test statistic 11.283, p = 0.001).

**Table 2:** Between group comparison of the number of qPCR positive dogs (Pos) and apparent pathogen prevalence (%) with 95% CI found for single infections at study baseline, 3-months, 6-months, and 8-months. Only one individual was found coinfected at both 6- and 8-months by *H.* *canis* and haemotropic mycoplasma*.*

Pathogen	Cohort (N)	Baseline (n = 40)		3-Months (n = 40)		6-Months (n = 40)		8-Months (n = 38)	
		Pos	% (CI)	Pos	% (CI)	Pos	% (CI)	Pos	% (CI)
*Anaplasma* *platys*	Detick	2	10 (3–30)	0	0 (0–16)	0	0 (0–16)	0	0 (0–16)
	Seresto^®^	0	0 (0–16)	0	0 (0–16)	0	0 (0–16)	0	0 (0–18)
	Both products	2	5 (1–17)	0	0 (0–9)	0	0 (0–9)	0	0 (0–9)
*Ehrlichia* *canis*	Detick	0	0 (0–16)	0	0 (0–16)	0	0 (0–16)	0	0 (0–16)
	Seresto^®^	1	5 (1–24)	0	0 (0–16)	0	0 (0–16)	0	0 (0–18)
	Both products	1	3 (0–13)	0	0 (0–9)	0	0 (0–9)	0	0 (0–9)
*Haemotropic* *mycoplasma*	Detick	1	5 (1–24)	3	15 (5–36)	4	20 (8–42)	3	15 (5–36)
	Seresto^®^	2	10 (3–30)	4	20 (8–42)	7	35 (18–57)	9	50 (29–71)
	Both products	3	8 (3–20)	7	18 (9–32)	11	28 (16–43)	12	32 (19–47)
*Babesia* *vogeli*	Detick	0	0 (0–16)	0	0 (0–16)	0	0 (0–16)	0	0 (0–16)
	Seresto^®^	0	0 (0–16)	0	0 (0–16)	0	0 (0–16)	0	0 (0–18)
	Both products	0	0 (0–9)	0	0 (0–9)	0	0 (0–9)	0	0 (0–9)
*Babesia* *gibsoni*	Detick	0	0 (0–16)	0	0 (0–16)	0	0 (0–16)	0	0 (0–16)
	Seresto^®^	0	0 (0–16)	0	0 (0–16)	0	0 (0–16)	0	0 (0–18)
	Both products	0	0 (0–9)	0	0 (0–9)	0	0 (0–9)	0	0 (0–9)
*Hepatozoon* *canis*	Detick	0	0 (0–16)	0	0 (0–16)	0	0 (0–16)	0	0 (0–16)
	Seresto^®^	1	5 (1–24)	1	5 (1–24)	1	5 (1–24)	1	6 (1—26)
	Both products	1	3 (0–13)	1	3 (0–13)	1	3 (0–13)	1	3 (0–13)
All pathogens	Detick	3	15 (5––36)	3	15 (5–36)	4	20 (8–42)	3	15 (5–36)
	Seresto^®^	4	20 (8–42)	5	25 (11–47)	8	40 (22–61)	10	56 (34–75)
	Both products	7	18 (9–32)	8	20 (11–35)	12	30 (18–45)	13	34 (21–50)

**Figure 1:**
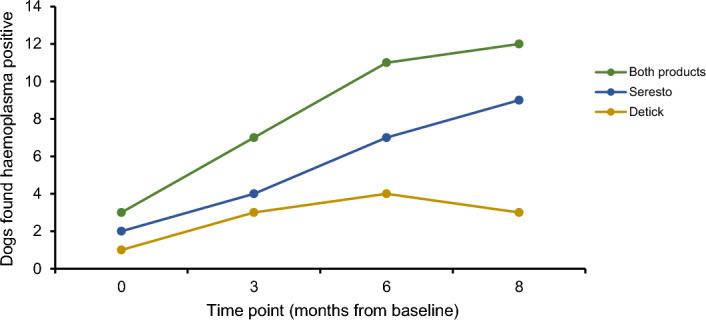
Change in the prevalence of haemoplasma infections in canines on the ectoparasiticides Detick, Seresto^®^, and the pooled results of dogs on both products, across an 8-month trial duration. Dots represent time points at which dogs were examined for ectoparasite presence and blood samples were collected and tested for pathogens.

Haemotropic mycoplasma infections increased over the course of this trial, particularly in the Seresto^®^ dog group (Fig. 1), whilst in the Detick group haemotropic mycoplasma infections increased from baseline to 3-months and then remained constant. The incidence of canine haemoplasma infection for dogs on Seresto^®^ was 44 per 100 dogs at risk per year, whilst for Detick it was 11 per 100 dogs at risk per year, when the number of new haemoplasma infections was pooled for dogs on both ectoparasiticide products the incidence was 26 per 100 dogs at risk per year. Chi-squared tests found no difference between the number of haemotropic mycoplasma infections in dogs on Seresto^®^ compared to those on Detick at all time points; baseline (χ^2^ test statistic 0.346, p = 0.556), 3-months (χ^2^ test statistic 0.157, p = 0.692), 6-months (χ^2^ test statistic 0.949, p = 0.330) and 8-months (χ^2^ test statistic 3.563, p = 0.059).

When the haemoplasma results were pooled from dogs on both ectoparasiticide products, dog sex was found to influence infection status at the 8-month timepoint, with male dogs more likely to be haemoplasma positive than female dogs at this time point (χ^2^ test statistic 3.970, p = 0.046). However, variables such as dog neutering status (χ^2^ test statistic 1.119, p = 0.290) and whether a dog was above or below two years of age (χ^2^ test statistic 0.002, p = 0.964) were not found to impact whether an individual was haemoplasma positive at the 8-month timepoint.

Sanger sequencing of a fragment of the haemotropic mycoplasma RNAse P gene using blood samples from the dogs found positive via qPCR within this study, returned a top hit in GenBank with *M.* *haemocanis* (100% identity match and query cover with accession no. AF407211.1). NCBI accession numbers for our sequences are OQ378200 to OQ378206.

## Discussion

This community trial provided extensive information on the transmission of haemotropic mycoplasmas between dogs within closed environments in the absence of ticks, fleas, and lice. Whilst this study demonstrated that the ectoparasiticide products Seresto^®^ and DeTick prevent canines from contracting confirmed VBPs, such as *Babesia* spp., *A.* *platys,* and *E.* *canis*, they did not prevent the contraction of haemoplasma infections in a country and local environment where these pathogens are highly endemic^13^. The large increase in haemoplasma infections detected over the course of this study demonstrates that both products are ineffective at preventing the transmission of such pathogens, likely due to them being communicable through mechanisms that are not reliant on a vector. Despite a report of vertical transmission from bitch to pup^35^ and other more circumstantial evidence of fighting-related transmission^33^, iatrogenic transmission through blood transfusion^34^ and a potential, but as yet unconfirmed role of arthropod vectors^2, 7, 26^, the mode(s) of canine haemotropic mycoplasma transmission is still not known. Here, we show clear evidence of non-vectorial canine haemoplasma transmission in the absence of vertical or transfusion-based infection, likely due to fighting between conspecifics in a closed environment. Prior data to indicate transmission of canine haemoplasmas via fighting has only been posited due to risk factor associations between dog aggression and haemotropic mycoplasma infection^33^, whilst this study builds upon such theories by providing more robust evidence.

The increase in haemotropic mycoplasma infections, particularly in the Seresto^®^ group, could in our context be a result of aggressive behaviours as dogs were kept communally and free to move in large enclosures where fighting was observed to be commonplace. The owner and carer for these dogs reported regular instances of canine aggression and fighting between individuals. This violent behaviour was observed more regularly in the group of dogs on Seresto^®^ (pers. comms. Georgia Kaczorowski, director of ‘House of Strays’), a fact that is further reflected in the breakage and removal of Seresto^®^ collars in this group due to antagonistic interactions between dogs.

When the results for haemoplasma infection between dogs on both ectoparasiticides were pooled and assessed at the study’s end, i.e., the 8-month time point, male dogs were found to have significantly higher levels of haemoplasma infection than female dogs. Ethological research has highlighted differences in tendencies to display aggressive behaviours between the sexes in canines, with male dogs found to display more aggression, particularly towards conspecifics outside of their household and dogs of the same sex^42–44^. Such findings may support the hypothesis that canine haemotropic mycoplasmas transmission may be occurring through fluid exchanges as a result of biting and fighting within our cohort of dogs.

In contrast, no differences were found between haemoplasma infection and dog age category and neutering status, whilst the limited variety of dog breeds within our study group prevented comparison with this variable. The absence of difference in infection levels between canine neutering status is notable given that there has been some research suggesting intact dogs may demonstrate more aggressive behaviours and initiate more fights with conspecifics than neutered dogs^43, 45^, although, other studies have found no such association, or that the inverse is true^46, 47^. In Asia, the neutering status of dogs was strongly associated with exposure to a range of zoonotic parasites, however, a possible explanation for such an association is that owners who have their dogs neutered may have better access to veterinary care, as opposed to those that leave their dogs intact^48^.

Whilst specific research on the transmission of canine haemoplasmas is lacking, some relevant data can be gleaned from a larger body of research investigating feline haemotropic mycoplasmas. In cats infected with *C.* M. turicensis, this pathogen’s DNA was detected in the saliva and faeces of the host early in the course of infection^38^, whilst *C.* M. haemominutum DNA has been detected from the salivary glands of infected cats^37^. The presence of haemoplasmas in saliva and faeces could provide a means by which these pathogens are transmitted horizontally, either during fighting or through social contacts^37, 38^. In addition, Museux et al. created a laboratory model to replicate possible haemoplasma transmission events through cat fighting and found that when as little as 10 μl of cat blood infected with *C.* M. turicensis was inoculated subcutaneously into a naïve cat it could produce an infection^49^. The possibility of such transmission modes being employed by haemoplasma species that are closely related to canine hemotropic mycoplasmas poses the possibility that similar processes could be occurring for these pathogens.

No concomitant rises in pathogens known to be transmitted by arthropod vectors was observed within this study, whilst no ectoparasites were ever found on dogs in either group, further supporting our conclusion that the rise in canine haemoplasma infections was not due to vectorial transmission. Other studies have found similar results when looking for correlations between ectoparasite infestations and canine haemoplasma infection, garnering data that does not suggest a transmission role is being played by arthropod vectors^31–33^. The possibility that canine haemoplasmas use other vectors, such as mosquitoes or sandflies, that were not presently investigated is possible, although there is limited data suggesting such arthropods can act as viable vectors for these pathogens, whilst ectoparasiticides like Seresto^®^ have been shown to be effective at preventing sandfly transmitted pathogens^50–52^.

Another putative explanation is that some dogs that tested positive for haemotropic mycoplasmas may have been pathogen carriers from the onset of the trial, whilst potentially fluctuating levels of parasitaemia meant that detection of these pathogens only occurred at certain time points. Such fluctuations in bacterial copy number, but no changes in the overall positivity status to haemoplasmas, have been observed before, in cats experimentally infected with *C.* M. turicensis^49^. This explanation could also potentially be the reason for the slight, but not statistically significant decrease in haemotropic mycoplasma infections observed from 6-months to 8-months in the group of dogs on Detick (one dog). Nonetheless, the sharp and statistically significant increase in the number of haemotropic mycoplasma positive dogs over time, together with a concomitant lack of detection of other VBPs and ectoparasites, strongly suggests that non-vectorial transmission of these pathogens is occurring.

Importantly, no treatment was initiated for haemotropic mycoplasmas until after the study had finished, hence the reduction in infections observed in the Detick group could either reflect a cure achieved by the dogs’ immune systems or a relative decrease in parasitaemia, making such infections temporarily undetectable by qPCR. However, the multiplex qPCR herein utilised has shown a very high analytical sensitivity of 5.2 × 10^–4^ fg/µl canine haemotropic mycoplasma DNA^53^, being suitable for the detection of animals with very low haemoplasma bacteraemia.

Environmental ectoparasite prevalence in Cambodia is known to be high with dogs on no ectoparasiticide product regularly found with tick and flea infestations, as well as high rates of VBP infection^13^. Therefore, the benefit and protection conferred by these ectoparasiticide products is great for those pathogens that are confirmed to be vectorially transmitted in countries such as Cambodia. Nonetheless, alternative measures need to be taken to prevent haemotropic mycoplasma infection, whilst further research on this group’s transmission is required to better unravel and understand the exact mechanism these pathogens harness to infect dogs. Our data is non-trivial given the potential pathogenesis canine haemotropic mycoplasmas can inflict on their hosts. Through cPCR and Sanger sequencing analysis we identified the haemoplasma species infecting our community dogs to be *M.* *haemocanis* which has been found to be highly prevalent in stray dogs from Cambodia before^13^. Species such as *M.* *haemocanis* and *C.* M. haematoparvum can generate a haemolytic anaemia with lethargy, fever, and icterus in infected dogs, whilst splenectomised or immunosuppressed individuals can show even more severe pathology that can lead to fatality^11, 54–56^. However, within this study no clinical abnormalities were observed between dogs infected versus those that were non-infected by canine haemoplasmas. In addition, some canine haemotropic mycoplasmas are zoonotic with one case in the literature identifying a *C.* M. haematoparvum, *A.* *platys* and *Bartonella* *henselae* coinfection from a symptomatic individual with pronounced disease^18^.

Overall, both Detick and Seresto^®^ collars showed a very high efficacy at preventing ectoparasite infestation and VBP infection, however, both products failed to prevent haemoplasma infections, suggesting that these pathogens are transmitted between dogs through a mechanism that does not involve ectoparasite vectors. Future work to unequivocally demonstrate whether alternative transmission modes are used by dog haemoplasmas, such as through fighting or social contact, is urgently warranted to assist in the development of guidelines that will prevent canine infections from this pathogen group. This data will garner valuable basic biology data on haemotropic mycoplasmas and could in turn shed light on other pathogenic species that infect different hosts including animals and humans, that to date have unknown modes of transmission^2, 19, 20, 25^.

## Methods

### Study design and study area

Ethical approval for this study was provided by the Office of Research Integrity and Ethics at the University of Melbourne, Australia, under ethics permit 1814620. This community trial was performed in accordance with the relevant guidelines and regulations including, but not limited to, those set by the University of Melbourne’s Office of Research Integrity and Ethics as well as those set by the Ministry of Agriculture, Forestry and Fisheries, Cambodia. Additionally, these methods are in accordance with ARRIVE guidelines.

This community trial was designed to compare the chemoprophylactic efficacy of two topical commercial acaricides; an imidacloprid 10% and flumethrin 4.5%, 8-month acting collar (Seresto^®^, Elanco) against a monthly spot-on containing [12% w/v] fipronil (Detick, Thailand), administered according to labelled instructions^40^. Both ectoparasiticide products act topically with the active compounds working from the subject’s skin or hair, hence they are designed to prevent vector feeding and reduce VBP transmission^40^.

A cohort of 40 dogs, with 20 in each treatment group (either Seresto^®^ or Detick), were enrolled from the ‘House of Strays’ dog shelter in Siem Reap, Cambodia (13° 38′ N, 103° 85′ E), an organisation supported by the ‘Animals of Our World’ charity (registration no. 1197372). Animals were deemed eligible for enrolment if they were over eight weeks of age and were clinically normal on physical examination, as determined by a qualified veterinarian. The composition of dogs in both treatment groups was similar with regard to sex, age, and breed (Table 1), however the group given Seresto^®^ had some individuals that had not been neutered (30%) whilst all dogs in the group given Detick were neutered. The only pure breed dog in our study cohort was a Pitbull.

All dogs had previously been administered the systemically-acting isoxazoline fluralaner as the formulation Bravecto^®^ (Merck Animal Health), however they were only enrolled into this study after the window of labelled efficacy for this product had ceased. Enrolled dogs were housed as two closed and separate populations of canines at the ‘House of Strays’ shelter which consisted of two large outdoor enclosures in which the groups were kept separate and unable to intermingle. Nonetheless, dogs within each group could mix and interact as they wished within the specific section of the shelter they were located in, which comprised of large expanses of grass, sand/dirt and some tree cover. Dogs in each group were kept in their respective separate enclosures at night. Within the grounds of the shelter, dogs from both groups were regularly exposed to ectoparasites such as ticks, fleas, and lice that could enter the environment or be brought in from local stray dogs and/or new arrivals to the shelter.

This trial was conducted from 09.12.2020 to 20.08.2021 with Seresto^®^ collars fitted and Detick applied at the start of trial. Seresto^®^ has a purported 8-month duration of efficacy, hence these products did not need to be replaced over the course of the 8-month trial unless a collar was inadvertently removed during fighting or play which occurred in the case of eight dogs given these collars over the course of this study. If prematurely removed a new collar was applied within 48 h. For the Detick group, this ectoparasiticide was reapplied monthly between the shoulder blades as per the manufacturer’s instructions. Follow-up time points were at 3-, 6-, and 8-months after study commencement, when canine sampling and relevant metadata were collected.

### Sampling and diagnostic methods

At enrolment, dogs were subject to a complete physical examination by the study’s veterinarians. Each animal’s age, sex, breed, neutering status, whether imported, prior ectoparasite and endoparasite control history as well as other ongoing medication details were recorded. The presence or absence of ectoparasites was also assessed. Two millilitres of blood sample were drawn via cephalic or jugular venipuncture from each dog into two EDTA tubes. Blood was subjected to an examination for TBPs using stained whole blood and buffy coat smears. The second tube of whole blood was frozen at − 20 °C and transported on ice to the Melbourne Veterinary School at the University of Melbourne.

At the Melbourne Veterinary School DNA extraction on blood was conducted using a DNeasy Blood & Tissue Kits (Qiagen, Hilden, Germany) and subject to a previously developed multiplex real-time (qPCR) assay for common canine VBPs for the region^53^. Data was collected on the positivity status for the pathogens *Anaplasma* *platys*, *Babesia* *gibsoni*, *Babesia* *vogeli*, *Ehrlichia* *canis,* canine haemotropic mycoplasmas and *Hepatozoon* *canis*. Sample and metadata collection at follow up was then conducted at 3-, 6- and 8-months after trial commencement and at all time points qPCR on blood-extracted DNA was carried out.

### Clinical and safety observations

At follow-up time points dogs were examined for the presence of ectoparasites for five minutes using visual observation and an ectoparasite comb with extra attention paid to common feeding sites of ticks e.g., head, ears, inter-digital regions. If ectoparasites were found, they were collected and preserved in 70% ethanol. Additionally, clinical signs consistent with VBP infection and potential adverse effects caused by their chemoprophylaxis treatment (e.g., dermatological changes at the site of application) were checked for. Individuals found positive to haemotropic mycoplasmas via qPCR during the trial were only treated at the end of the study i.e., after 8-months post commencement with a course of doxycycline 10 mg/kg SID for 28 days. For the individuals found positive to *A.* *platys* at the study’s baseline a 14-day course of doxycycline was initiated, whilst for the single *E.* *canis* positive dog found at baseline a 28-day course was commenced. No treatment was commenced for any dog found positive to a protozoan pathogen.

### Conventional PCR and Sanger sequencing analyses

The utilised multiplex qPCR for canine VBP was only able to detect whether a sample was positive for canine haemotropic mycoplasmas at genus level. Hence, two conventional PCR (cPCR) assays were employed to provide species-level classification; one targeting a 600 bp stretch of the 16S ribosomal RNA (16S rRNA) gene of haemoplasmas^57^ and another targeting a 165 bp stretch of the haemotropic mycoplasma ribonuclease P gene (RNase P)^58^. All cPCRs were conducted using Taq 2X Master Mix (New England Biolabs, MA, USA) according to the published methods^57, 58^. Positive amplicons were cleaned using ExoSAP-IT™ PCR Product Cleanup Reagent (Thermo Fisher Scientific, MA, USA) and sent to Macrogen (Seoul, South Korea) for Sanger sequencing. Results were run through BLASTn, to compare them to existing GenBank reference sequences.

### Data handling and statistics

To identify differences between the number of newly acquired haemotropic mycoplasma infections between dogs on Detick and Seresto^®^, univariate analyses were conducted using 2 × 2 chi-square tests to assess whether there were statistically significant differences between the counts of haemoplasma qPCR test positives and negatives at all time points (p-value < 0.05). To identify associations between the number of newly acquired haemoplasma infections versus confirmed VBP infections, such as *A.* *platys*, *E.* *cani*s and *H.* *canis,* 2 × 2 chi-square tests were also conducted, pooling infections from dogs on both ectoparasiticides together. Univariate analysis carried out via chi-squared tests were also used to assess canine haemoplasma infection status with dog sex, age, and neutering status at 8-months, irrespective of ectoparasiticide treatment.

Incidence rates for canine haemoplasma infection were determined by the number of new infections, i.e., the number of infections at 8-months minus the baseline infections, divided by the population at risk, i.e., total number of dogs that made it through the study minus those that were infected at baseline. In addition, the 95% confidence intervals (CIs) for haemotropic mycoplasma prevalence at all time points were calculated using the Wilson score interval via the open-source software Epitools (https://epitools.ausvet.com.au).

## Data Availability

NCBI accession numbers for our Sanger sequences (Partial RNAse P gene of *M.* *haemocanis*) are OQ378200 to OQ378206. All datasheets, metadata and qPCR results are available from the authors upon request.
